# Supplementary material for: GroEL/ES chaperonin unfolds then encapsulates a nascent protein on the ribosome
Source: Nat Commun. 2025 Nov 13;16:9976. doi: 10.1038/s41467-025-64968-w (PMC12615815; doi:10.1038/s41467-025-64968-w)
Supplement: Supplementary file 1 — Supplementary Information [file 41467_2025_64968_MOESM1_ESM.pdf]

Supplementary information

GroEL/ES chaperonin unfolds then encapsulates a nascent protein on the ribosome

Alžběta Roeselová[1], Sarah L. Maslen[2], Jessica Zhiyun He[3], Gabija Jurkeviciute[3], Aleksandra Pajak[1], J. Mark Skehel[2], Radoslav I. Enchev[3], David Balchin[1]*

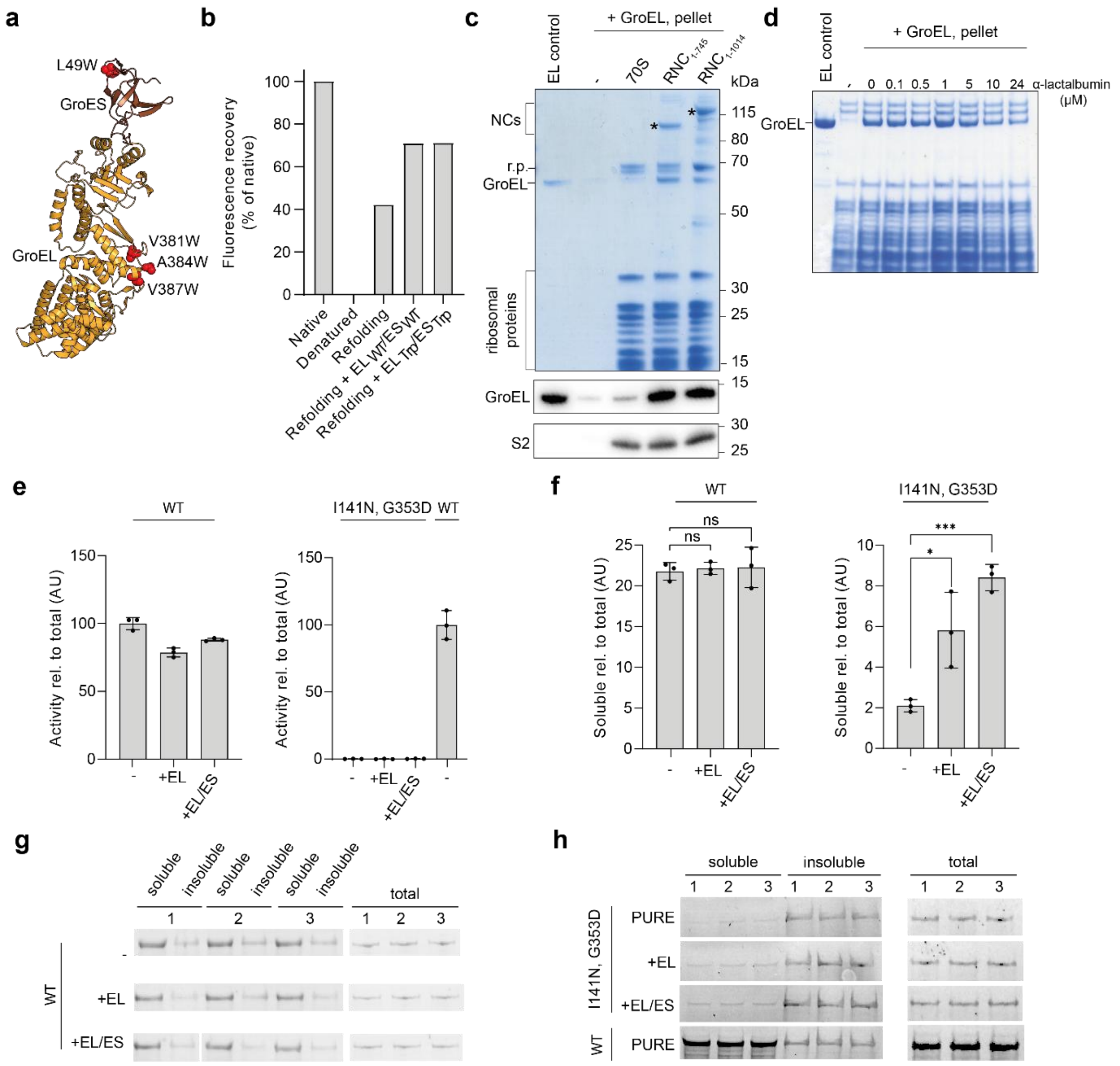
a
L49W
GroES
GroEL
V381W
A384W
V387W
b
Fluorescence recovery (% of native)
Native
Denatured
Refolding
Refolding + EL WT/ES WT
Refolding + EL Trp/ES Trp
c
EL control
+ GroEL, pellet
70S
RNC1-745
RNC1-1014
kDa
NCs
r.p.
GroEL
ribosomal proteins
S2
d
EL control
+ GroEL, pellet
α-lactalbumin (μM)
GroEL
e
WT
I141N, G353D
Activity rel. to total (AU)
+EL
+EL/ES
f
WT
I141N, G353D
Soluble rel. to total (AU)
ns
+EL
+EL/ES
g
soluble
insoluble
total
WT
+EL
+EL/ES
h
soluble
insoluble
total
I141N, G353D
PURE
+EL
+EL/ES
WT

Supplementary Figure 1. GroEL co-sediments with RNCs. a Cartoon representation of a GroEL subunit bound to GroES (PDB: 7VWX) with residues 381, 384 and 387 in GroEL (yellow) and residue 49 in GroES (brown) shown as red spheres. These positions are mutated to Trp in the GroEL/ES molecules used throughout this study. **b** GFP refolding by GroEL/ES. GFP fluorescence (λ_{ex} = 470 nm, λ_{em} = 515 nm) was recorded 20 minutes after 1.25 µM HCl-denatured eGFP was diluted 100-fold into denaturing buffer (30 mM HCl), refolding buffer, or refolding buffer with 4 µM GroEL (WT or Trp - V381W, A384W, V387W) and 8 µM GroES (WT or Trp - L49W), all in the presence of 2 mM ATP. The fluorescence signal was normalised to the fluorescence signal of native eGFP, to quantify the fluorescence recovery. **c** Coomassie-stained SDS-PAGE of the resuspended ribosomal pellet from a co-sedimentation assay. Prior to sedimentation, GroEL was incubated with either buffer (-), empty ribosomes (70S), RNC_{1-745}, or RNC_{1-1014}. RNCs were purified from *Δtig* cells. Bands corresponding to the NCs (*), GroEL, and ribosomal proteins (r.p.) are indicated. Purified GroEL was loaded in the first lane for reference (EL control). Bottom: immunoblot of a replicate SDS-PAGE gel probed using antibodies against GroEL and ribosomal protein S2. The experiment was repeated three times with similar results. **d** A post-translational client does not efficiently outcompete GroEL binding to RNCs. Coomassie-stained SDS-PAGE of the resuspended ribosomal pellet from a co-sedimentation assay. Prior to sedimentation, 5 µM $RNC_{1-510mut}$ was incubated with buffer (-) or 5 µM GroEL. Where indicated, reactions were supplemented with varying concentrations (0-24 µM) of reduced α-lactalbumin. The experiment was repeated twice with similar results. **e** Activity of β-galactosidase detected in in vitro translation reactions expressing wild-type (WT) or destabilised (I141N, G353D) full-length β-galactosidase. Prior to protein expression, reactions were supplemented with RNC low-salt buffer (-), 1 µM GroEL (+EL), or 1 µM GroEL and 3 µM GroES (+EL/ES). Activity levels are expressed normalised to the total amount of full-length β-galactosidase produced in the reactions. Data are presented as mean ± SD, n = 3 independent translation reactions. **f** Amount of full-length β-galactosidase detected in the soluble fractions of in vitro translation reactions expressing wild-type (WT) or destabilised (I141N, G353D) full-length β-galactosidase. Prior to expression, in vitro reactions were supplemented with RNC low-salt buffer (-), 1 µM GroEL (+EL), or 1 µM GroEL and 3 µM GroES (+EL/ES). Amounts of soluble protein are expressed normalised to the total amount of full-length β-galactosidase produced in the reactions. Data are presented as mean ± SD, n = 3 independent translation reactions. ns - $p>0.05$; * - $p < 0.05$, *** - $p < 0.001$, one-way ANOVA with Dunnett's multiple comparisons. **g** SDS-PAGE gels scanned for fluorescent signal detecting the FluoroTect labelled full-length β-galactosidase band at ~115 kDa in soluble, insoluble and total fractions of in vitro translation reactions expressing wild-type (WT) full-length β-galactosidase. Prior to expression, in vitro reactions were supplemented with RNC low-salt buffer (-), 1 µM GroEL (+EL), or 1 µM GroEL and 3 µM GroES (+EL/ES). Data from 3 independent translation reactions (1,2,3) are shown. **h** SDS-PAGE gels scanned for fluorescent signal detecting the FluoroTect labelled full-length β-galactosidase band at ~115 kDa in soluble, insoluble and total fractions of in vitro translation reactions expressing wild-type (WT) or destabilised (I141N, G353D) full-length β-galactosidase. Prior to expression, in vitro reactions were supplemented with RNC low-salt buffer (-), 1 µM GroEL (+EL), or 1 µM GroEL and 3 µM GroES (+EL/ES). Data from 3 independent translation reactions (1,2,3) are shown. Source data are provided as a Source Data file.

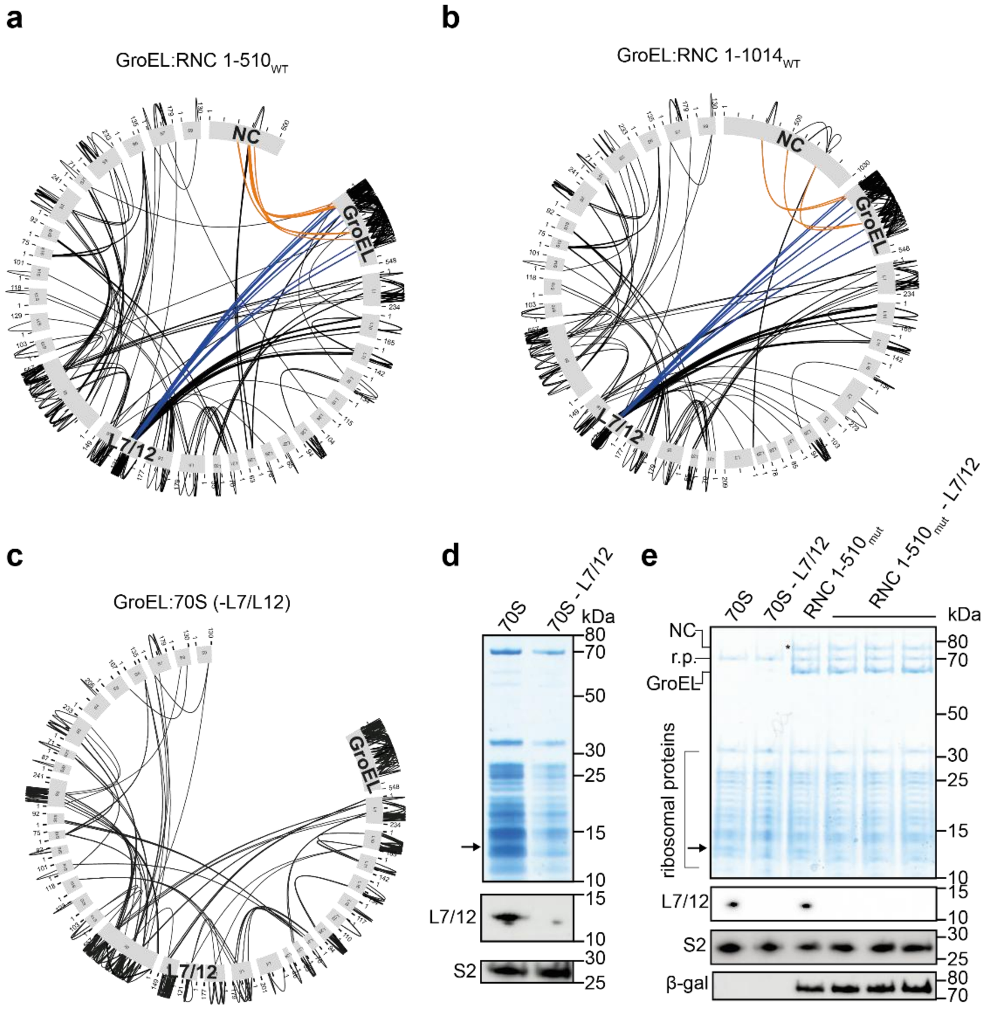

Supplementary Figure 2. GroEL crosslinking to empty ribosomes and RNCs. a Map of crosslinks between GroEL and $RNC_{1\text{-}510WT}$. Crosslinks between GroEL and the NC (orange) or L7/L12 (blue) are highlighted. **b** Map of crosslinks between GroEL and $RNC_{1\text{-}1014}$. Crosslinks between GroEL and the NC (orange) or L7/L12 (blue) are highlighted. **c** Map of crosslinks between GroEL and empty 70S ribosomes depleted of L7/L12. **d** Selective removal of L7/L12 from ribosomes. Top: Coomassie-stained SDS-PAGE of complete (70S) and L7/L12-depleted (70S-L7/12) ribosomes. The position of L7/12 is indicated by a black arrow. Bottom: Immunoblot of a replicate SDS-PAGE gel probed using antibodies against ribosomal proteins L7/12 and S2. The experiment was repeated twice with similar results. **e** Removing L7/L12 from $RNC_{1\text{-}510mut}$ does not prevent GroEL binding. Top: Coomassie-stained SDS-PAGE of the resuspended ribosomal pellet from a co-sedimentation assay. Prior to sedimentation, GroEL was incubated with either empty ribosomes (70S), 70S ribosomes after depletion of the ribosomal stalk (70S-L7/12), $RNC_{1\text{-}510mut}$ (RNC 1-510_{mut}), or RNC 1-510_{mut} after depletion of the ribosomal stalk (RNC 1-510_{mut}-L7/12). The experiment was repeated twice with similar results. The pelleting assay for the last condition (RNC 1-510_{mut}-L7/12) was conducted in triplicate. Bands corresponding to the NCs (*), GroEL, and ribosomal proteins (r.p.) are indicated. Bottom: Immunoblot of a replicate SDS-PAGE gel probed using antibodies against β-galactosidase and ribosomal proteins L7/12 and S2. Source data are provided as a Source Data file and in Supplementary Data 3.

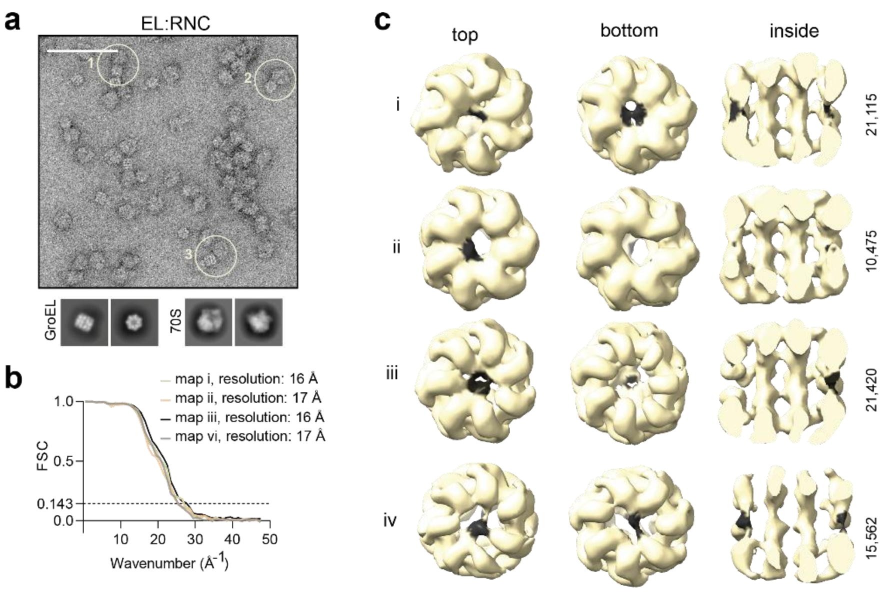

Supplementary Figure 3. nsEM of GroEL:RNC$_{1\text{-}510}$ complexes. a Top: Negative stain electron microscopy (nsEM) micrographs of uncrosslinked GroEL:RNC$_{1\text{-}510}$ complexes. The scale bar corresponds to 100 nm. Examples of GroEL positioned near ribosomes are circled (1-3). 500 micrographs were collected. Bottom: 2D class averages of GroEL and 70S ribosomes. **b** Fourier Shell Correlation (FSC) plots for reconstructions obtained from the uncrosslinked (maps i and ii) and DSBU-crosslinked (maps iii and iv) complexes. **c** 3D reconstructions of uncrosslinked (i, ii) and DSBU-crosslinked (iii, iv) GroEL: RNC$_{1\text{-}510}$ complexes from nsEM. Density not accounted for by the solved structure of GroEL (PDB: 5W0S) is coloured black. The number of particles contributing to each reconstruction is given on the right.

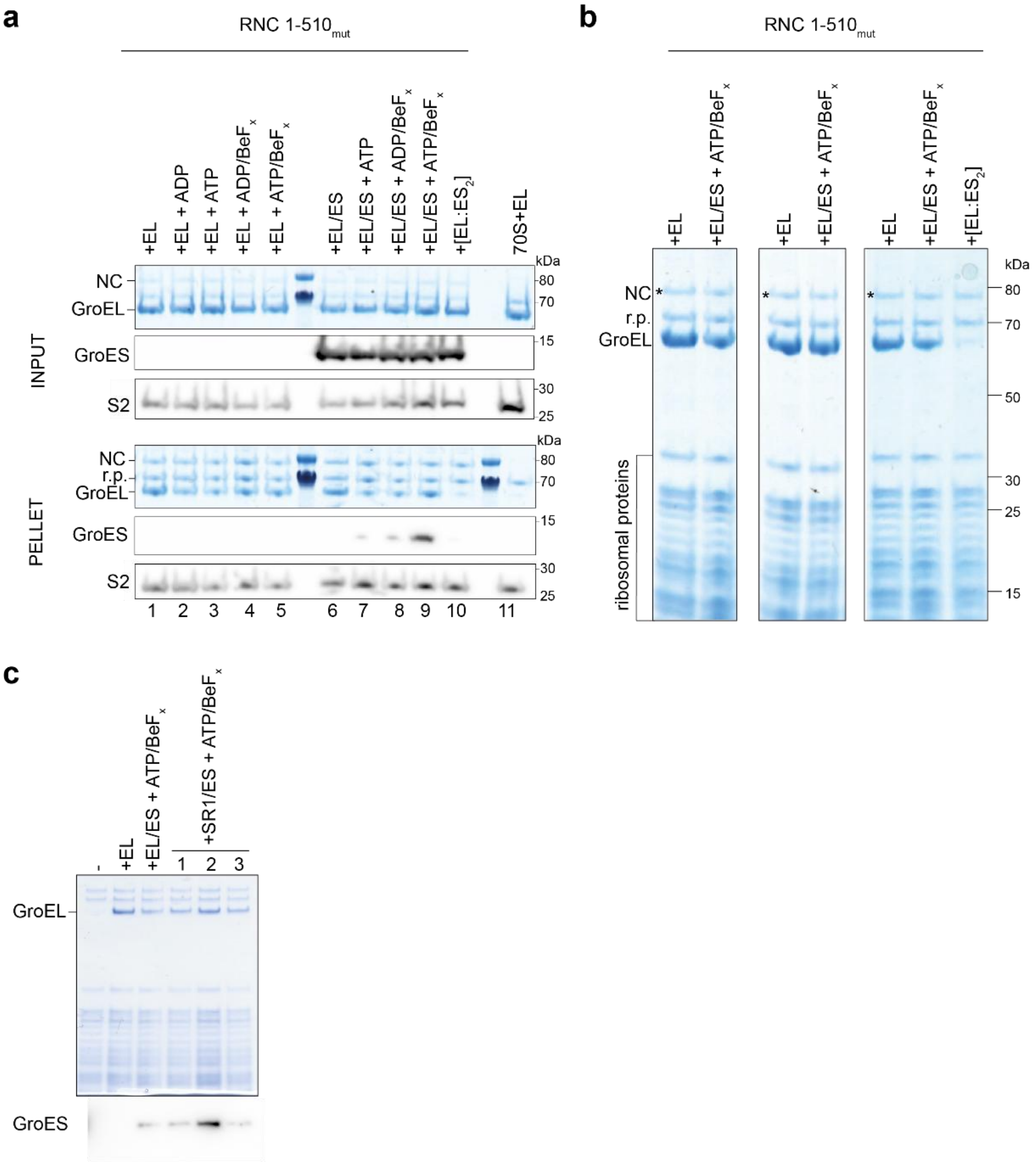
a
RNC 1-510mut
+EL
+EL + ADP
+EL + ATP
+EL + ADP/BeFx
+EL + ATP/BeFx
+EL/ES
+EL/ES + ATP
+EL/ES + ADP/BeFx
+EL/ES + ATP/BeFx
+[EL:ES2]
70S+EL
kDa
NC
GroEL
GroES
S2
INPUT
NC
r.p.
GroEL
GroES
S2
PELLET
80
70
15
30
25
1 2 3 4 5 6 7 8 9 10 11
b
RNC 1-510mut
+EL
+EL/ES + ATP/BeFx
+[EL:ES2]
NC
r.p.
GroEL
ribosomal proteins
kDa
80
70
50
30
25
15
c
-
+EL
+EL/ES + ATP/BeFx
+SR1/ES + ATP/BeFx
1 2 3
GroEL
GroES

Supplementary Figure 4. **GroEL/ES binding to $RNC_{1\text{-}510mut}$ in the presence of different nucleotides. a** Effect of different nucleotides on the stability of GroEL:RNC complexes. Coomassie-stained SDS-PAGE and immunoblot analysis of co-sedimentation assays of GroEL/ES with $RNC_{1\text{-}510mut}$ incubated with different nucleotides. Both input (top) and pellet (bottom) fractions are shown. Prior to sedimentation, the RNC was incubated with GroEL either in low-salt RNC buffer (1), or with additional 1 mM ADP (2), ATP (3), ADP/BeF_x (4) or ATP/BeF_x (5). Alternatively, the RNC was incubated with GroEL/ES in low-salt RNC buffer (6) or with additional 1 mM ATP (7), ADP/BeF_x (8) or ATP/BeF_x (9). As controls, the RNC was incubated with a pre-formed complex of EL:ES_2 (10) in the presence of ATP/BeF_x, or GroEL was incubated with empty 70S ribosomes (11). Any nucleotide and metal salts were present in the binding buffer as well as the sucrose cushion and wash buffers. Bands corresponding to the NCs (*),GroEL, and ribosomal proteins (r.p.) are highlighted. Below each Coomassie-stained gel are immunoblots from the same gel probed using antibodies against GroES and ribosomal protein S2. The experiment was repeated twice with similar results. **b** GroEL remains bound to RNCs upon addition of GroES and ATP/BeF_x. Coomassie-stained SDS-PAGE of resuspended ribosomal pellets from co-sedimentation assays of GroEL with $RNC_{1\text{-}510mut}$. Where indicated, RNCs were incubated with GroEL, or GroEL, GroES and ATP/BeF_x. As a control, GroEL, GroES and ATP/BeF_x were pre-mixed to form symmetrically closed complexes before adding to RNCs (+$[EL:ES]_2$). Bands corresponding to the NCs (*), GroEL, and ribosomal proteins (r.p.) are highlighted. Each lane corresponds to an independent co-sedimentation assay. The experiment was repeated twice with similar results. **c** GroES can bind a complex between GroEL single-ring (SR1) and $RNC_{1\text{-}510mut.}$ Coomassie-stained SDS-PAGE of resuspended ribosomal pellets from co-sedimentation assays of GroEL with $RNC_{1\text{-}510mut}$. Where indicated, RNCs were incubated with GroEL, or GroEL, GroES and ATP/BeF_x. Assays were performed with either WT GroEL (EL) or the single-ring mutant (SR1). The experiment was repeated twice with similar results. Cosedimentation with SR1 was performed in triplicate. Below, an immunoblot of the same gel using an antibody against GroES. Source data are provided as a Source Data file and in Supplementary Data 2.

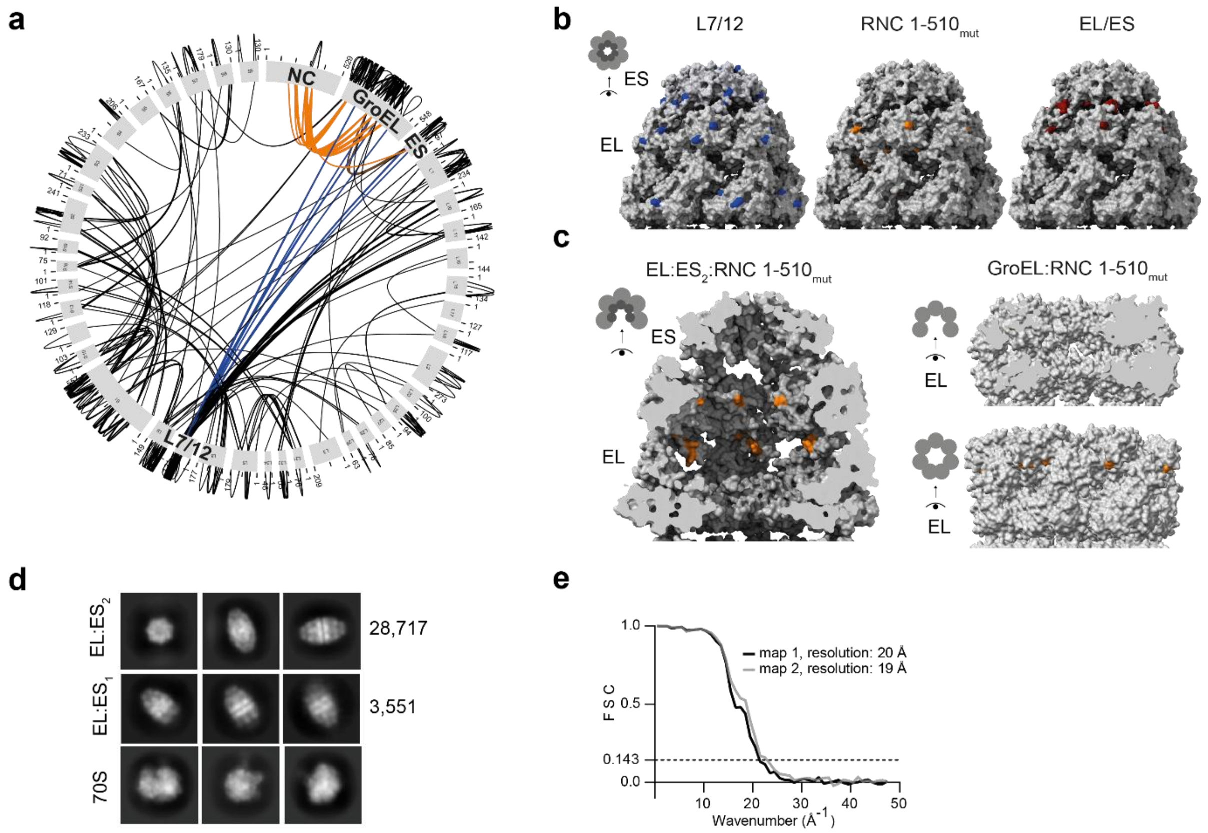

Supplementary Figure 5. Structural characterisation of GroEL:ES$_2$:RNC complex. a Map of crosslinks between GroEL/ES and RNC$_{1\text{-}510mut}$. Crosslinks between GroEL and GroES (brown), from GroEL/ES to the NC (orange), and from GroEL/ES to L7/L12 (blue) are highlighted. **b** Crosslink sites are mapped onto the structures of the ATP/BeF$_x$-stabilised EL:ES$_2$ complex (PDB:7VWX), showing the outer surface. Residues are separated according to whether they crosslink to L7/L12 (blue), the NC (orange), or connect GroEL and GroES (brown). **c** Change in accessibility of GroEL residues upon GroES binding. Left: inner surface of the GroEL/ES cavity (left, PDB:7VWX). Right: inner (top) and outer (bottom) surfaces of apo-GroEL (PDB: 5W0S). Residues which crosslinked to the NC in the EL:ES$_2$:RNC complex but not in the GroEL:RNC complex are shown in orange. **d** 2D class averages for double-capped GroEL (EL:ES$_2$), single-capped GroEL (EL:ES$_1$) and 70S ribosomes, from nsEM of the GroEL/ES:RNC complex. **e** Fourier Shell Correlation (FSC) plots for reconstructions obtained from nsEM analysis of EL:ES$_2$:RNC complexes. Source data are provided in Supplementary Data 3.

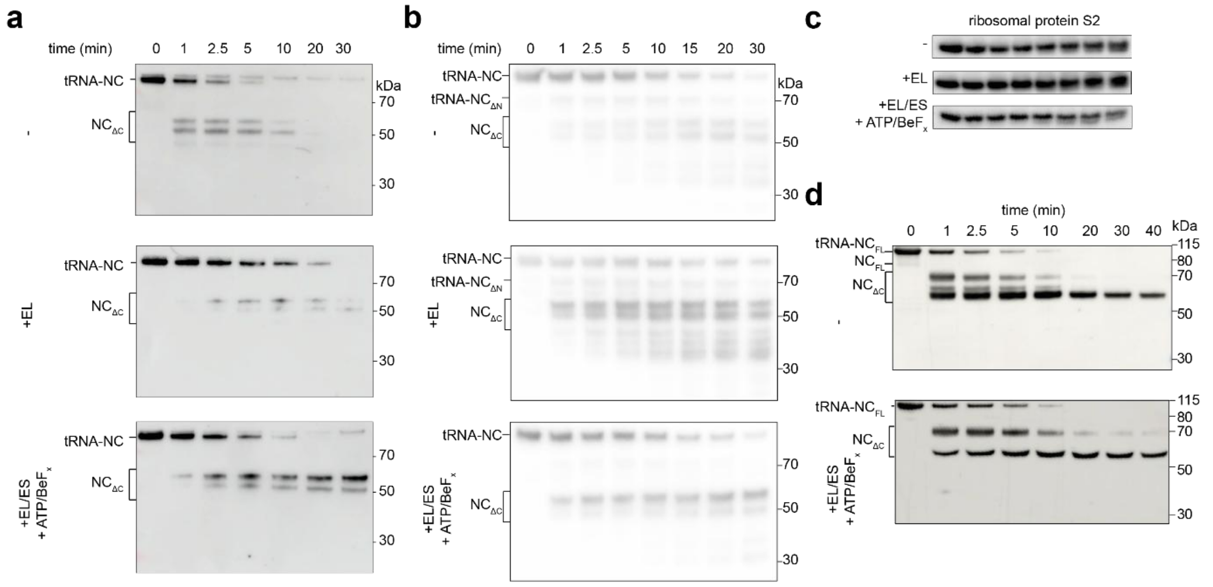

Supplementary Figure 6. Limited proteolysis of $RNC_{1\text{-}510mut.}$ a Replicate of limited proteolysis experiment shown in Figure 6a, using an antibody raised against the N-terminus of β-galactosidase. The experiment was repeated twice with similar results. **b** As in Figure 6, except GroEL/ES:RNC complexes were isolated by sedimentation through a sucrose cushion, prior to treatment with proteinase K. The experiment was repeated twice with similar results. **c** Loading control for limited proteolysis experiment shown in (B). Immunoblots were probed using an antibody against ribosomal protein S2. **d** Immunoblot membranes shown in Figure 6b probed with an antibody raised against the N-terminus of β-galactosidase. Source data are provided as a Source Data file

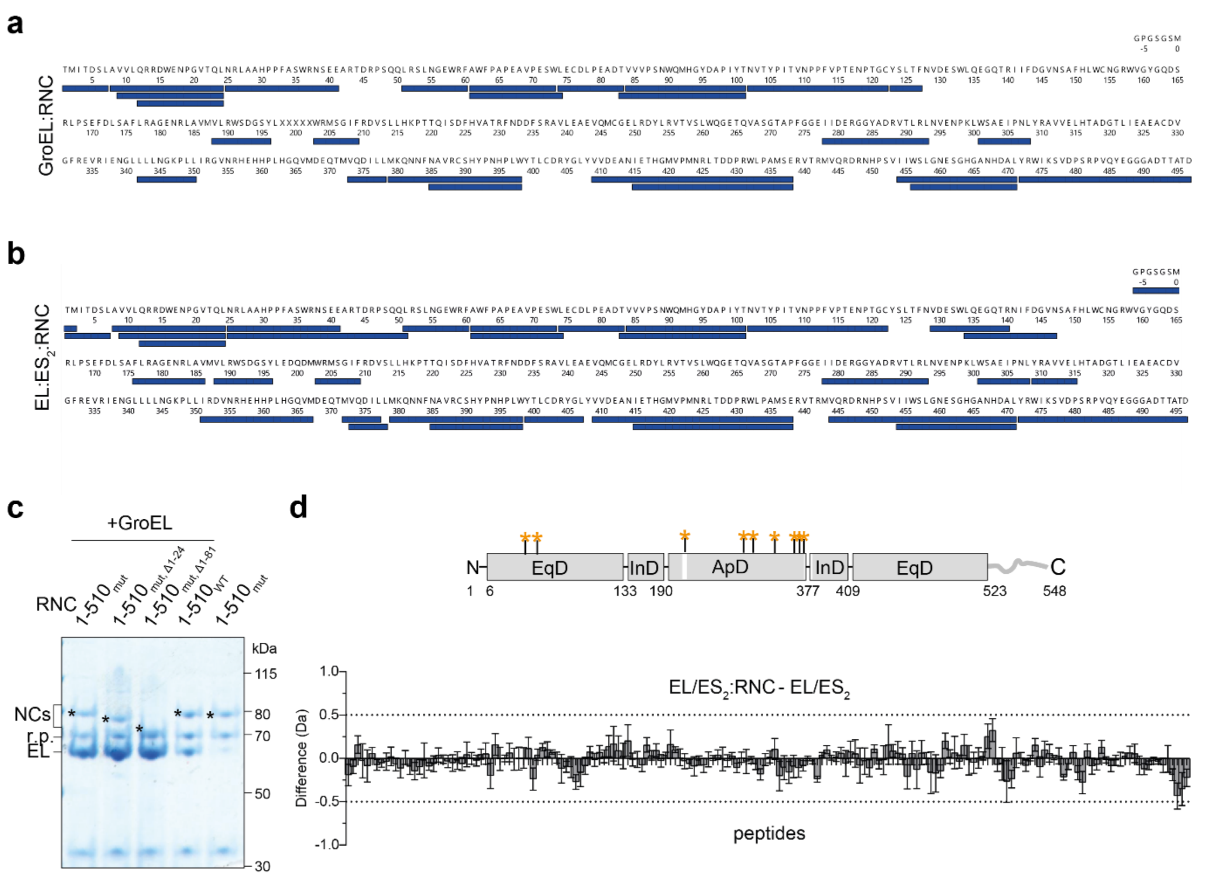

Supplementary Figure 7. GroEL/ES binding to RNCs. **a** Peptide coverage map of the NC in GroEL:RNC$_{1\text{-}510mut}$ complex. **b** Peptide coverage map of the NC in EL:ES$_2$ RNC$_{1\text{-}510mut}$ complex. **c** GroEL binding is unaffected by deleting residues 1-81 of NC$_{1\text{-}510mut.}$ Coomassie-stained SDS-PAGE of the resuspended ribosomal pellet from a co-sedimentation assay. Prior to sedimentation, GroEL was incubated with either wild-type RNC$_{1\text{-}510}$ (1-510$_{WT}$), RNC$_{1\text{-}510mut}$ (1-510$_{mut}$), or RNC$_{1\text{-}510mut}$ lacking residues 1-24 (1-510$_{mut\ Δ1\text{-}24}$) or 1-81 (1-510$_{mut\ Δ1\text{-}81}$). The final lane contains purified RNC 1-510$_{mut}$ without additional GroEL. Bands corresponding to the NCs (*), GroEL, and ribosomal proteins (r.p.) are indicated. The experiment was repeated twice with similar results. **d** Protection of GroEL upon binding GroES RNC$_{1\text{-}510mut}$. Difference in deuterium uptake after 100 s between isolated EL:ES$_2$ and EL:ES$_2$:RNC$_{1\text{-}510mut}$, both stabilised by ATP-BeF$_{x.}$ Values are plotted for individual GroEL peptides. Negative values indicate less deuteration of a peptide in when the RNC is bound. Data are presented as mean ± SD, n = 3 independent labelling reactions. Source data are provided as a Source Data file and in Supplementary Data 1.

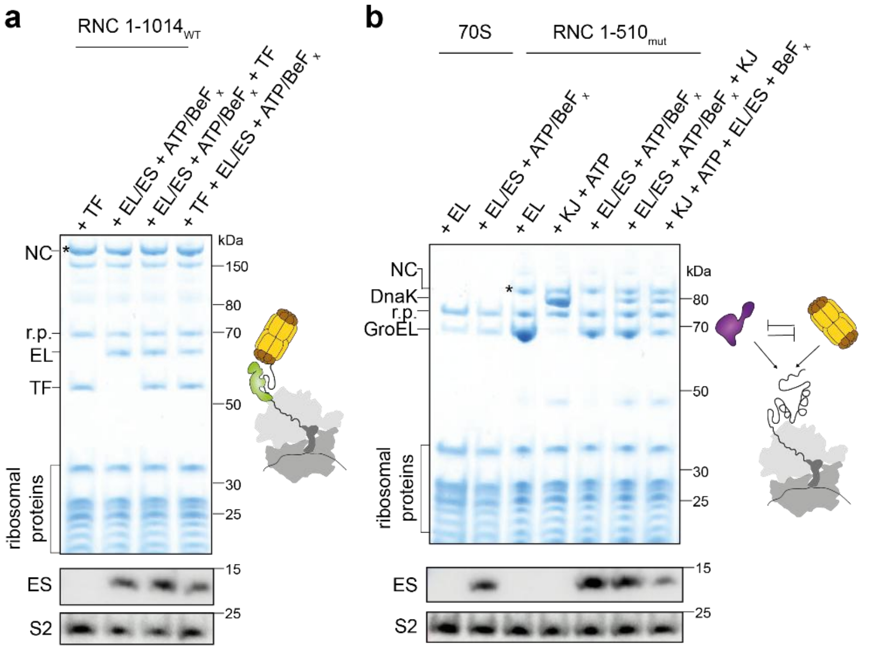

Supplementary Figure 8. Coordination of GroEL/ES with Trigger factor and DnaK. a TF and GroEL/ES do not compete for binding long NCs. Top: Coomassie-stained SDS-PAGE of the resuspended ribosomal pellet from co-sedimentation assays. Prior to sedimentation, $RNC_{1\text{-}1014}$ was incubated with Trigger factor (+TF), GroEL with GroES and ATP/BeF_x (+EL/ES), or a combination of the above in the specified order. Bands corresponding to the NC (*), TF, GroEL, and ribosomal proteins (r.p.) are indicated. Bottom: Immunoblot from an equivalent SDS-PAGE gel, probed against GroES and ribosomal protein S2. The experiment was repeated twice with similar results. **b** DnaK and GroEL/ES compete for binding NCs. Top: Coomassie-stained SDS-PAGE of the resuspended ribosomal pellet from co-sedimentation assays. Prior to sedimentation, empty ribosomes (70S) or $RNC_{1\text{-}510mut}$ were incubated with GroEL (+EL), DnaK with DnaJ (+KJ), GroEL with GroES and ATP/BeF_x (+EL/ES), or a combination of the above in the specified order. Bands corresponding to the NC (*), DnaK, GroEL, and ribosomal proteins (r.p.) are indicated. Bottom: Immunoblot from an equivalent SDS-PAGE gel, probed against GroES and ribosomal protein S2. The experiment was repeated twice with similar results. Source data are provided as a Source Data file.
