# Supplementary material for: GroEL/ES chaperonin unfolds then encapsulates a nascent protein on the ribosome
Source: Nat Commun. 2025 Nov 13;16:9976. doi: 10.1038/s41467-025-64968-w (PMC12615815; doi:10.1038/s41467-025-64968-w)
Supplement: Supplementary file 2 — Description of Additional Supplementary Files [file 41467_2025_64968_MOESM2_ESM.pdf]

Description of Additional Supplementary Files

File name: Supplementary Data 1
Description: HDX-MS data.

File name: Supplementary Data 2
Description: Proteomic analysis of resuspended pellets from co sedimentation assays.

File name: Supplementary Data 3
Description: XL-MS data.

File name: Supplementary Data 4
Description: List of DNA constructs and recombinant protein sequences.
