# Supplementary material for: GroEL/ES chaperonin unfolds then encapsulates a nascent protein on the ribosome
Source: Nat Commun. 2025 Nov 13;16:9976. doi: 10.1038/s41467-025-64968-w (PMC12615815; doi:10.1038/s41467-025-64968-w)
Supplement: Supplementary file 7 — Reporting Summary [file 41467_2025_64968_MOESM7_ESM.pdf]

nature portfolio

Corresponding author(s): David Balchin

Last updated by author(s): Sep 11, 2025

Reporting Summary

Nature Portfolio wishes to improve the reproducibility of the work that we publish. This form provides structure for consistency and transparency in reporting. For further information on Nature Portfolio policies, see our Editorial Policies and the Editorial Policy Checklist.

Statistics

For all statistical analyses, confirm that the following items are present in the figure legend, table legend, main text, or Methods section.

n/a	Confirmed	
☐	☒	The exact sample size (*n*) for each experimental group/condition, given as a discrete number and unit of measurement
☐	☒	A statement on whether measurements were taken from distinct samples or whether the same sample was measured repeatedly
☐	☒	The statistical test(s) used AND whether they are one- or two-sided<br>*Only common tests should be described solely by name; describe more complex techniques in the Methods section.*
☒	☐	A description of all covariates tested
☒	☐	A description of any assumptions or corrections, such as tests of normality and adjustment for multiple comparisons
☐	☒	A full description of the statistical parameters including central tendency (e.g. means) or other basic estimates (e.g. regression coefficient) AND variation (e.g. standard deviation) or associated estimates of uncertainty (e.g. confidence intervals)
☐	☒	For null hypothesis testing, the test statistic (e.g. *F*, *t*, *r*) with confidence intervals, effect sizes, degrees of freedom and *P* value noted<br>*Give P values as exact values whenever suitable.*
☒	☐	For Bayesian analysis, information on the choice of priors and Markov chain Monte Carlo settings
☒	☐	For hierarchical and complex designs, identification of the appropriate level for tests and full reporting of outcomes
☒	☐	Estimates of effect sizes (e.g. Cohen's *d*, Pearson's *r*), indicating how they were calculated

Our web collection on statistics for biologists contains articles on many of the points above.

Software and code

Policy information about availability of computer code

Data collection	HDX-MS data were collected using a Synapt G2Si (Waters). Proteomics data were collected using a Lumos Tribrid Orbitrap. XLMS data were collected using a Orbitrap Exploris 480. NsEM data were collected using a FEI Tecnai Spirit 120 kV TEM.
Data analysis	PLGS 3.0 (Waters) and Dynamx 3.0 (Waters) were used for analysis of HDX-MS data. Proteomics data were analysed using Maxquant 2.5 and Perseus 1.6.2.3. XLMS data were analysed using MeroX 2.0. NsEM data were processed using cryoSPARC. Models were docked into NsEM maps using UCSF ChimeraX 1.8. Western blots were analysed in ImageJ. Other data were analysed using Graphpad Prism 9.

For manuscripts utilizing custom algorithms or software that are central to the research but not yet described in published literature, software must be made available to editors and reviewers. We strongly encourage code deposition in a community repository (e.g. GitHub). See the Nature Portfolio guidelines for submitting code & software for further information.

Data

Policy information about availability of data

All manuscripts must include a data availability statement. This statement should provide the following information, where applicable:

- Accession codes, unique identifiers, or web links for publicly available datasets
- A description of any restrictions on data availability
- For clinical datasets or third party data, please ensure that the statement adheres to our policy

HDX-MS data are available in Supplementary Data 1, quantitative proteomics in Supplementary Data 2, and XL-MS data in Supplementary Data 3. The mass spectrometry proteomics data have been deposited to the ProteomeXchange Consortium via the PRIDE partner repository with the following dataset identifiers:

Composition of ribosomal pellets: PXD054251 [https://proteomecentral.proteomexchange.org/cgi/GetDataset?ID=PXD054251]
HDX-MS: PXD054376 [https://proteomecentral.proteomexchange.org/cgi/GetDataset?ID= PXD054376]
XL-MS: PXD054379 [https://proteomecentral.proteomexchange.org/cgi/GetDataset?ID=PXD054379]
Source data are provided with this paper.

Research involving human participants, their data, or biological material

Policy information about studies with human participants or human data. See also policy information about sex, gender (identity/presentation), and sexual orientation and race, ethnicity and racism.

Reporting on sex and gender	Not applicable
Reporting on race, ethnicity, or other socially relevant groupings	Not applicable
Population characteristics	Not applicable
Recruitment	Not applicable
Ethics oversight	Not applicable

Note that full information on the approval of the study protocol must also be provided in the manuscript.

Field-specific reporting

Please select the one below that is the best fit for your research. If you are not sure, read the appropriate sections before making your selection.

[x] Life sciences [ ] Behavioural & social sciences [ ] Ecological, evolutionary & environmental sciences

For a reference copy of the document with all sections, see nature.com/documents/nr-reporting-summary-flat.pdf

Life sciences study design

All studies must disclose on these points even when the disclosure is negative.

Sample size	No statistical methods were used to calculate sample sizes. Biochemical experiments were repeated at least three times, and in some cases using independent protein purifications. 3-4 replicates of HDX-MS data were collected to account for technical variability in pipetting, considering prior evidence in the literature supporting the high technical reproducibility of these experiments, and consistent with community guidelines for HDX MS (Masson, G. R. et al. Recommendations for performing, interpreting and reporting hydrogen deuterium exchange mass spectrometry (HDX-MS) experiments. Nat Methods 16, 595–602 (2019)). In some cases, replicate data were also acquired for independent purifications of the protein complexes to assess biological variability. For other experiments (PK assays, IVTs), the high reproducibility and large effect sizes indicate that the number of replicates was sufficient. Experiments were repeated 2-3 times. For NsEM, 30-40,000 particles were used to generate each 3D map. No sample size calculation was performed for the EM data; the sample size is sufficient for the resolution of the map reported here.
Data exclusions	For NsEM, particles were excluded based on 2D/3D classification according to standard practice in the EM community.
Replication	All experiments except NsEM and XL-MS were confirmed using replicate measurements. EM single particle analysis involves averaging many independent particles. XL-MS involves analysis of many independent crosslinks per sample.
Randomization	No randomization was performed, as samples were not grouped.
Blinding	No blinding was performed, as samples were not grouped. Moreover, MS data were analysed using standard pipelines which minimise the possibility of subjective interpretation.

Reporting for specific materials, systems and methods

We require information from authors about some types of materials, experimental systems and methods used in many studies. Here, indicate whether each material, system or method listed is relevant to your study. If you are not sure if a list item applies to your research, read the appropriate section before selecting a response.

Materials & experimental systems

n/a	Involved in the study	
☐	☒	Antibodies
☒	☐	Eukaryotic cell lines
☒	☐	Palaeontology and archaeology
☒	☐	Animals and other organisms
☒	☐	Clinical data
☒	☐	Dual use research of concern
☒	☐	Plants

Methods

n/a	Involved in the study	
☒	☐	ChIP-seq
☒	☐	Flow cytometry
☒	☐	MRI-based neuroimaging

Antibodies

Antibodies used	anti-GroEL (abcam, ab82592),<br>rabbit anti-GroES (Enzo, ADI-SPA-210-D)<br>rabbit anti-S2 (Antibodies-Online, ABIN2938988)<br>rabbit anti-L7/L12 (abcam, ab225681)<br>Anti-β-galactosidase (abcam, ab221199)<br>Anti-β-galactosidase (abcam, ab106567)<br>HRP-conjugated goat anti-rabbit (abcam, ab205718),<br>anti-mouse (abcam, ab205719)<br>anti-chicken (abcam, ab97135)<br>All primary antibodies were diluted 1:1000 and secondary antibodies were diluted 1:10,000
Validation	10.1016/j.cels.2017.08.016<br>/10.1073/pnas.94.20.10967<br>10.1016/j.celrep.2022.110663<br>Also the current manuscript, using purified Bgal, GroEL, GroES and 70S ribosomes.

Plants

Seed stocks	Not applicable
Novel plant genotypes	Not applicable
Authentication	Not applicable
